# Differentiated thyroid cancer in patients with resistance to thyroid hormone syndrome. A novel case and a review of the literature

**DOI:** 10.3389/fmolb.2014.00010

**Published:** 2014-09-02

**Authors:** João Vinagre, Fátima Borges, António Costa, Maria Inês Alvelos, Glaúcia Mazeto, Manuel Sobrinho-Simões, Paula Soares

**Affiliations:** ^1^Institute of Molecular Pathology and Immunology of the University of PortoPorto, Portugal; ^2^Institute of Biomedical Sciences Abel Salazar (ICBAS)Porto, Portugal; ^3^Department of Endocrinology, Diabetes and Metabolism, Centro Hospitalar do PortoPorto, Portugal; ^4^Department of Surgery 2, Centro Hospitalar do PortoPorto, Portugal; ^5^Hospital das Clínicas de BotucatuSão Paulo, Brazil; ^6^Medical Faculty of the University of PortoPorto, Portugal; ^7^Department of Pathology, Centro Hospitalar São JoãoPorto, Portugal

**Keywords:** resistance to thyroid hormone, thyroid cancer, mPTC, *THRB*, *BRAF*

## Abstract

Resistance to thyroid hormone (RTH) represents a syndrome in which patients present elevated circulating thyroid hormones in the presence of non-suppressed TSH. We report a novel case where a patient with RTH presented a differentiated thyroid cancer. A19 year-old female had been referred due to thyroid disease that disclosed features characteristic of a RTH. During the follow up it was detected a follicular tumor that led to the recommendation for thyroid surgical ablation, where an incidental papillary thyroid microcarcinoma (mPTC) was found. The increase of thyroglobulin (TG) levels following thyroid removal referred the patient for radioiodine treatment. Post-treatment, it was detected jugular adenopathies and the patient was subjected to cervical lymph node drainage where metastases of the mPTC were found. RTH syndrome was confirmed by the detection of a *THRB* germline mutation. A *BRAF* mutation was also found in the mPTC but not detected in the follicular adenoma or normal adjacent tissue. The young age of the patient, the rarity of *BRAF* mutations in childhood and the high dissemination of the malignancy, lead us to the speculation that increased TSH stimulation in a RTH background and oncogenic activation of *BRAF* could have served as (co) drivers and might have triggered an advanced stage of the neoplastic disease. These findings together with a review of published cases add novel information to the management of RTH patients with differentiated thyroid cancer.

## Introduction

Thyroid hormone receptors (TRs) belong to the nuclear receptor superfamily that regulates the activity of thyroid hormone, triiodothyronine (T_3_), responsible for growth, differentiation and metabolism. The T_3_-binding receptors are presented in four major isoforms: TRα1, TRβ 1, TRβ 2, and TRβ 3. These receptors are codified by *THRA* and *THRB* genes present on chromosomes 17 and 3, respectively (Yen, 2001). TRs are able to bind to specific DNA motifs on the promoters of T_3_-target genes, thyroid hormone responsive elements (TREs), leading to its transcription activation or repression (Guigon and Cheng, 2009).

Mutations in *THRB* gene lead to a decreased tissue responsiveness to thyroid hormones and cause Resistance to thyroid hormone (RTH) syndrome. Most mutations cluster in a hotspot area encoding the T_3_-binding domain that spans exons 8–10 (Yen, 2001). However, a RTH phenotype can also manifest in absence of *THRB* alterations (Beck-Peccoz et al., 2006). The disease is normally inherited in an autosomal dominant manner, and the mutant receptors usually exert a dominant negative activity over the wild-type receptors interfering with receptor dimerization and binding to TREs (Guigon and Cheng, 2009). Clinically, the hallmark of RTH patients is the presence of high levels of free thyroid hormones in the presence of measurable TSH. The clinical phenotype of these patients is variable with most patients presenting mild to moderate symptoms, but the most common finding is the presence of goiter (Refetoff and Dumitrescu, 2007).

In the last years, a few cases of RTH and differentiated thyroid cancer have been reported (Taniyama et al., 2001; Kim et al., 2010; Paragliola et al., 2011; Ramos-Prol et al., 2013; Unluturk et al., 2013). We present a novel case in which mPTC was associated with a *THRB* germline mutation. Due to the unexpected progression of the mPTC we decided to investigate further the case looking for molecular alterations relevant for the development, in this particular case, of a metastatic disease.

## Materials and methods

### Informed consent

All procedures followed were in accordance with the national ethical standards previously approved by Local Ethical Review Committees. Informed consent was obtained from the patient for being included in the study.

### Case report

**Figure 1:**
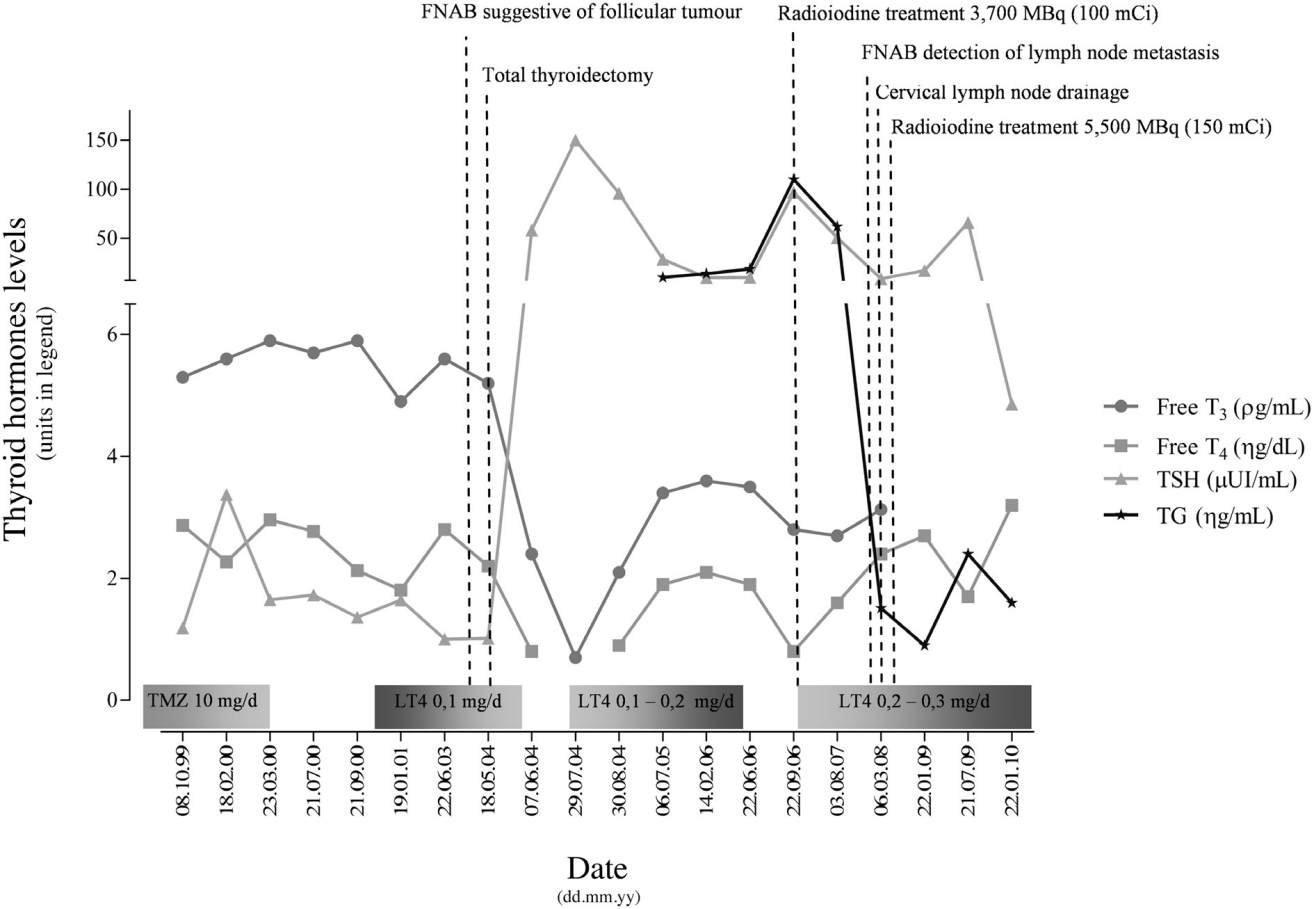
**Major events, clinical measurements and therapy management of the patient referring the period on first appearance until the last appointment**. Abbreviations: TMZ, Thiamazole; LT4, Levothyroxine.

In late 1999, a 19-year-old female was referred to the Endocrinology Department due to thyroid disease. On physical examination, she presented goiter, a heart rate of 80 beats per min, no Graves' ophthalmopathy and no hand tremor although it was observed palmar hyperhidrosis. The patient had no signs of emotional disturbances, learning disabilities or mental retardation, though it was noticeable a hyperactive behavior. Clinical measurements (Figure 1) revealed out of range levels of circulating free thyroxine (T_4_) (2.87 η g/dL, normal range 0.89–1.76 η g/dL) and free triiodothyronine T_3_ (5.3 ρ g/mL, normal range 2.0–4.2 ρ g/mL) with the presence of non-suppressed TSH (1.188 μUI/mL, normal range 0.4–4.4 μUI/mL). Antithyroid antibodies were also measured and were at non-detectable levels discarding thyroid autoimmune disease. The patient had been prescribed with antithyroid therapy (thiamazole 10 mg per day orally) to control the mild hyperthyroidism. Novel evaluation presented once more abnormal high levels of free T_3_ and T_4_ in the presence of TSH. These results pointed to a case of central hyperthyroidism. To exclude the presence of a pituitary adenoma, the patient was subjected to a CT scan and α-GSU was assayed. The coronal CT scan revealed a normal gland without focal hypodensity or characteristics suggestive of a pituitary adenoma, and α-GSU dosage was within normal levels. Taking all this into consideration a diagnosis of RTH was proposed.

RTH diagnosis was later (2008) confirmed by mutation analysis of the *THRB* gene that disclosed a germline missense mutation. Both parents were tested for the mutation and no alterations were detected indicating a *de novo* origin.

**Figure 2:**
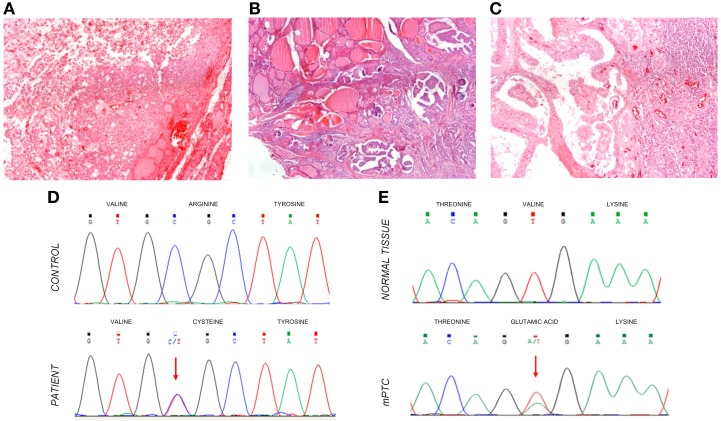
**Haematoxylin-eosin stained histological sections of a follicular adenoma, initially detected in the patient (A); a papillary microcarcinoma (B); and metastasis of the mPTC in a lymph node following the cervical drainage (C)**. The total magnification of the sections is 200X. Molecular genotyping of the samples detected the presence of a *THRB* germline mutation detected in DNA extracted from blood cells **(D)**. Somatic mutation analysis disclosed the presence of a *BRAF* V600E alteration in the mPTC (**E**, bottom sequence) whereas a wild-type *BRAF* sequence was present in the normal adjacent tissue (**E**, top sequence).

The patient was referred for monitoring and was followed on a regular basis. In mid 2003, it was noticeable a thyroid enlargement and palpation exam of the thyroid gland indicated the presence of solid nodules. Ultrasound imaging confirmed solid, well circumscribed nodules on the left lobe and a smaller one on the right side. Fine needle aspiration biopsy (FNAB) of the nodules indicated follicular tumor(s) and the patient was recommended for total thyroidectomy. Surgical thyroid ablation was performed in May 2004. Histological examination of the left lobe revealed the presence of follicular patterned nodules, the biggest one, a fetal follicular adenoma with 15 mm (Figure 2A). The right lobe presented a 4 mm mPTC (Figure 2B). Post-surgical TSH level was 58 μUI/mL, and the patient started replacement therapy with levothyroxine. The patient presented a body weight averaging 56 Kg, so it started to receive an initial dose of 0.1 mg/day of levothyroxine (≈1.7 μg/Kg). The starting dose was based on the recommended dosage for management of hypothyroidism according to the American Thyroid Association (ATA) guidelines. Since RTH patients require higher doses of replacement hormone, medication was meant to be adjusted periodically (using the TSH as a guideline) in order to lower TSH levels and maintain normal levels of thyroid hormone. However, during the adjustment the patient started to present tremors, weight loss, irritability and sudation. These symptoms led to the decision of slowing the gradually introduction of supraphysiological doses for full replacement. Due to the mPTC incidental finding, thyroglobulin (TG) levels started to be checked. In 2006, TG levels were slowly increasing and the patient was submitted to radioiodine treatment 3700 MBq (100 mCi). The patient remained under regular appointments for assessment and TG levels kept being monitored. During 2007, an imagiological study detected homolateral jugular adenopathies. Evaluation of FNAB cytologic slides revealed metastases of papillary thyroid carcinoma (PTC). The patient was subjected to cervical lymph node drainage. Histological examination found metastases of PTC in 7 out of 17 lymph nodes (Figure 2C). Another treatment with 5500 MBq (150 mCi) was prescribed. Following the second treatment with radioiodine, TG values dropped to residual levels. In 2010, the patient appears to be free of metastatic disease, although treatment with levothyroxine is still implemented and has reached 0.3 mg/day (≈5.35 μg/Kg) in order to control TSH levels. The previous symptoms presented by the patient were attenuated with the introduction of the beta-blocker, prescribed mainly to control tachycardia that started to manifest as a consequence of the levothyroxine dosage.

### Thyroid antibodies and hormone levels quantification

Measurements of thyroid autoantibodies were performed by radioimmunoassay (RIA) (Brahms Diagnostica, Hennigsdorf, Germany). The thyroid hormone levels were measured by electrochemiluminescence immunoassay (ECLIA) (Roche Diagnostic GmbH, Mannheim, Germany). Both determinations were carried out according to the manufacturer specifications.

### Genetic analysis

Briefly, following whole blood DNA extraction, PCR amplification was performed and *THRB* gene was screened for mutations. Whole blood genomic DNA extraction was prepared using NucleoSpin Tissue Kit (Macherey-Nagel, Düren, Germany). Extraction of tumor and adjacent tissue DNA from paraffin-embedded material was performed using the Invisorb spin tissue mini kit (Invitek, Berlin, Germany). Tumor and normal tissue samples were screened for *TRHB BRAF*, *N* and *H-RAS* and *TERT* promoter mutation. Amplifications were done using conditions described elsewhere (Trovisco et al., 2005; Rocha et al., 2007; Vinagre et al., 2013). PCR products were then purified and bidirectional sequenced on an ABI Prism 3130 *xl* Automatic sequencer (Perkin-Elmer, Foster City, USA) using the ABI Prism Dye Terminator Cycle sequencing kit (Perkin-Elmer).

## Results

Direct sequencing of *THRB* exon 9 showed a germline heterozygous mutation of a single base change in codon 320, Figure 2D. The mutation leads to an Arginine substitution by a Cysteine (R320C). The mutation found had been previously associated to a RTH case and functional characterization revealed that it induces a reduction of approximately 50% in T_3_ binding affinity (Burman et al., 1992). Additionally we screened the parents for the *THRB* mutation but it was not found, reflecting a *de novo* origin. Mutational analysis of *BRAF* (Figure 2E) in the mPTC and the lymph node metastasis revealed a Valine to Glutamic acid substitution at codon 600 (V600E). The *BRAF* mutation was not found in the normal adjacent tissue or in the follicular adenoma. No mutations were found in *TERT* promoter.

## Discussion

RTH corresponds to a syndrome of reduced responsiveness of the target tissues to thyroid hormone. Refetoff et al. initially described it in 1967 (Refetoff et al., 1967). In a daily practice, the main evidence of RTH still reflects the initial observations, presence of elevated levels of circulating free T_4_ and T_3_ along with normal or high levels of non-suppressed TSH (Beck-Peccoz et al., 2006). In the present case, the patient presented this trait, Figure 1, which pointed to a case of central hyperthyroidism, and RTH syndrome was thought to be the cause. Clinical manifestations of RTH can be very heterogeneous (Refetoff and Dumitrescu, 2007), among the most frequent symptoms of RTH the patient only exhibited, besides the abnormal hormonal levels, goiter and a hyperactivity disorder. In RTH the detection of parents and siblings with identical abnormal thyroid hormone and TSH levels were proposed by some authors as enough to provide the diagnosis. However, cases of RTH likely due to *de novo* mutations, as is the present case, have been identified in 28% of families with the condition (Refetoff and Dumitrescu, 2007).

With the identification of *THRB* gene mutations by Sakurai et al. (1989), the majority of the cases of RTH syndrome could be assigned to thyroid hormone nuclear receptor alterations. Nonetheless, 15% of individuals who present a RTH phenotype do not carry mutations in *THRB* gene. These are designated as “non-thyroid receptor RTH,” and can present mutations in selenocysteine insertion sequence-binding protein 2 (*SBP2*) or in monocarboxylate transporter 8 (*MCT8*) genes (Refetoff and Dumitrescu, 2007). Our patient presented a “classic” RTH associated with a *THRB* mutation (R320C) that had been previously characterized and resulted in decreased T_3_ binding affinity (Burman et al., 1992), therefore confirming the advanced diagnosis. Since we found a mutation in *THRB* we did not screen *SBP2* and *MCT8* for mutations. The *THRB* mutations detected in RTH patients cluster mainly in the last 4 exons (7–10) that code for the hinge region and the T_3_ hormone ligand-binding domain of the receptor. These regions correspond to the carboxyl-terminus of the thyroid hormone receptor β. The majority of these mutations result in the modification of the ligand-binding domain site that does not allows the binding of the thyroid hormone and thus does not function as a proper receptor. Additionally to the reduction in T_3_-binding, it can also lead to impairment in transactivation.

Somatic mutations of *THRB* have been previously described in hepatocellular, renal cell carcinoma and other cancers but do not present a pivotal role in human thyroid carcinogenesis as we and others, were unable to detect *THRB* mutations in human follicular thyroid adenomas, PTC or FTC (Rocha et al., 2007; Ramos-Prol et al., 2013). Increased frequency of thyroid cancer was not commonly observed or was not reported in RTH patients. More recently, cases with thyroid cancer in RTH patients started to be reported and so far there are already 7 cases in the literature (Taniyama et al., 2001; Kim et al., 2010; Paragliola et al., 2011; Ramos-Prol et al., 2013; Unluturk et al., 2013); reviewed in Table 1. *THRB* mutations can lead to increased TSH levels by ultimately disrupting the hypothalamic-pituitary-thyroid axis tight regulation and TSH has long been known to increase adenylate cyclase activity leading to cAMP production and promoting cell growth (Dumont et al., 1992).

**Table 1:** **Review of known cases of RTH patients with differentiated thyroid cancer**.

**References**	**Age**	**Gender**	**Tumor**			**Treatment of**	**DTC follow-up**
			**Histology**	**Size**	**Genotyping**	**1. RTH**	
						**2. DTC**	
Taniyama et al., 2001	46	F	FvPTC	5 mm	*THRB* R249Q	1. Methimazole	NA
						2. Subtotal thyroidectomy	
Kim et al., 2010	38	F	PTC	4 mm^*^	*THRB* M310T	1. Levothyroxin	NA
						2. Total thyroidectomy	
Paragliola et al., 2011	48	M	PTC	24 mm	ND	1. Levothyroxin	9.5 years
						2. Total thyroidectomy	In remission
Paragliola et al., 2011	63	M	PTC	6 mm	*THRB* P453T	1. Levothyroxin	5 years
						2. Total thyroidectomy	In remission
Unluturk et al., 2013	29	F	PTC	8 mm	*THRB* T334C	1. Levothyroxin	21 years
						2. Subtotal thyroidectomy (1st approach)	In remission
						2. Completion thyroidectomy and radioiodine (2nd approach)	
Unluturk et al., 2013	33	F	PTC	12 mm	ND	1. Levothyroxin	1 year
						2. Total thyroidectomy and radioiodine	In remission
Ramos-Prol et al., 2013	9	F	PTC	24 mm^*^	*THRB* R243W	1. TRIAC and levothyroxine	2 years
						2. Total thyroidectomy and radioiodine	In remission
Current case	19	F	PTC	4 mm	*THRB* R320C	1. Levothyroxin	6 years
						2. Total thyroidectomy and radioiodine	In remission

RTH, Resistance to thyroid hormone syndrome; DTC, Differentiated thyroid cancer; FvPTC, Follicular variant of papillary thyroid carcinoma; PTC, Papillary thyroid carcinoma; ND, Not detected; NA, Not available; TRIAC, Triiodothyroacetic acid;

* *multifocal tumors*.

In the present case, an occult mPTC was found after thyroidectomy. The mPTC is a very common condition, being a frequent incidental finding in thyroid gland removal for other reasons, or in autopsy (Rosai et al., 2003). Although mPTC's usually show an indolent behavior, some (3–15%) have the capability to spread to regional lymph nodes, and exceptionally metastasize to other organs (Simpson and Albores-Saavedra, 2007). Generally, mPTC developed in adolescence and young adulthood tend to present a growing rate similar to the thyroid gland, unless additional events occur and cause an increase in their growth rate (Rosai et al., 2003). It is well established that neck lymph node metastases are common in young individuals with papillary thyroid carcinoma and this fact contributes to the idea that thyroid cancer arising in a young patient is associated with a higher aggressiveness. However, most of the evidence in record concerns clinically evident papillary carcinomas. Regarding our case, we are referring an incidental papillary microcarcinoma, where it was demonstrated that age was not a significant factor affecting lymph node metastasis (Gulben et al., 2008). So, in addition to the continual thyrotrophic TSH stimulation we sought to identify what could have been an additional event(s) in our case. We evaluated the frequent oncogenic mutation in genes *N-RAS*, *H-RAS*, and *BRAF* as potential relevant factors additionally to the TSH stimulation. We also analyzed *TERT* promoter mutations, a recent molecular change associated to thyroid tumors (Vinagre et al., 2013). Molecular analysis determined the presence of a *BRAF* mutation in the mPTC as well as in the respective metastases. Such finding fits with the papillary growth pattern, and is in agreement with the high frequency of *BRAF* mutations (43%) we detected in a series of mPTC displaying papillary pattern (Trovisco et al., 2005). Conversely, *BRAF* mutation is associated with older patients, and tends to be rare in young adulthood PTC tumors (Trovisco et al., 2005; Fugazzola et al., 2006). Together, the young age of the patient and the high dissemination of the malignancy, lead us to suggest that increased TSH stimulation in a RTH background and oncogenic activation of *BRAF* could have served as (co) drivers. We are now aware that the management of the patient was not the most adequate as TSH levels still remained elevated although it is well established that maintenance of a suppression of TSH is challenging in this cases (Unluturk et al., 2013). The continual TSH stimulation as a consequence of the inadequate hormone replacement might have been a major tumor growth contributor. This stimulation probably causes the enlargement of the thyroid gland and the goitrogenic effect seen in patients with RTH syndrome and TSH-secreting pituitary adenomas. Cases of pituitary TSH-secreting adenomas in association with thyroid cancer have also been reported in the literature (for a review, please refer to reference Unluturk et al., 2013). These associations between TSH stimulation and thyroid cancer could also be supported by the studies referring the association between thyroid cancer and serum TSH concentration, and the possible role of TSH in the progression of differentiated thyroid cancer (for a thorough review, see reference Boelaert, 2009). Additional interesting observation refers to the possible role of thyroid-stimulating antibodies simulating TSH effect in Grave's disease associated thyroid cancer. Although it remains a matter of controversy, studies have suggested an increase in aggressiveness (frequent distant metastases) of PTC and FTC in Grave's disease patients (Filetti et al., 1988; Yano et al., 2007).

Summing up, we speculate that the concurrence of *TRHB* mutation, high TSH levels and *BRAF* mutation might have acted toward the malignant transformation and the aggressiveness of the mPTC in the present case. All the other cases reviewed did not present tumor genotyping so we cannot infer about the *BRAF* role in the other reported patients. Aggressive treatment can be an option to prevent tumor recurrence and persistence in the absence of an ideal TSH suppression, as was the case we report. Finally, we believe this subject will continue to be debatable, and new cases and novel presentations should be reported as so far the outcome of the reported cases have not been unfavorable and they will bring novel knowledge and information to this issue.

### Conflict of interest statement

The authors declare that the research was conducted in the absence of any commercial or financial relationships that could be construed as a potential conflict of interest.
